# Hyperbaric oxygen therapy for long COVID (HOT-LoCO), an interim safety report from a randomised controlled trial

**DOI:** 10.1186/s12879-023-08002-8

**Published:** 2023-01-20

**Authors:** Anders Kjellberg, Adrian Hassler, Emil Boström, Sara El Gharbi, Sarah Al-Ezerjawi, Jan Kowalski, Kenny A. Rodriguez-Wallberg, Judith Bruchfeld, Marcus Ståhlberg, Malin Nygren-Bonnier, Michael Runold, Peter Lindholm

**Affiliations:** 1 Department of Physiology and Pharmacology, Karolinska Institutet, Stockholm, Sweden; 2 Perioperative Medicine and Intensive Care, Medical Unit Intensive Care and Thoracic Surgery, Karolinska University Hospital, Stockholm, Sweden; 3 Medical Unit Emergency Medicine, Karolinska University Hospital, Stockholm, Sweden; 4 JK Biostatistics AB, Stockholm, Sweden; 5 Department of Oncology and Pathology, Karolinska Institutet, Stockholm, Sweden; 6 Division of Gynaecology and Reproduction, Department of Reproductive Medicine, Karolinska University Hospital, Stockholm, Sweden; 7 Division of Infectious Diseases, Department of Medicine Solna, Karolinska Institutet, Stockholm, Sweden; 8 Department of Infectious Diseases, Karolinska University Hospital, Stockholm, Sweden; 9 Department of Medicine Solna, Karolinska Institutet, Stockholm, Sweden; 10 Medical Unit Cardiology, Heart, Vascular and Neuro Theme, Karolinska University Hospital, Stockholm, Sweden; 11 Division of Physiotherapy, Department of Neurobiology, Care Sciences and Society, Karolinska Institutet, Stockholm, Sweden; 12 Women’s Health and Allied Health Professionals Theme, Medical Unit Occupational Therapy and Physiotherapy, Karolinska University Hospital, Stockholm, Sweden; 13 Department of Medicine Solna, Respiratory Medicine Unit, Karolinska Institutet, Stockholm, Sweden; 14 Department of Respiratory Medicine and Allergy, Karolinska University Hospital, Stockholm, Sweden; 15 Division of Hyperbaric Medicine, Department of Emergency Medicine, University of California San Diego, La Jolla, CA 92093 USA

**Keywords:** Long COVID, Post COVID condition, HRQoL, RCT, Clinical trial, Hyperbaric oxygen, HBOT, Safety

## Abstract

**Background:**

With ~ 50 million individuals suffering from post-COVID condition (PCC), low health related quality of life (HRQoL) is a vast problem. Common symptoms of PCC, that persists 3 months from the onset of COVID-19 are fatigue, shortness of breath and cognitive dysfunction. No effective treatment options have been widely adopted in clinical practice. Hyperbaric oxygen (HBO_2_) is a candidate drug.

**Methods:**

The objective of this interim analysis is to describe our cohort and evaluate the safety of HBO_2_ for post covid condition. In an ongoing randomised, placebo-controlled, double blind, clinical trial, 20 previously healthy subjects with PCC were assigned to HBO_2_ or placebo. Primary endpoints are physical domains in RAND-36; Physical functioning (PF) and Role Physical (RP) at 13 weeks. Secondary endpoints include objective physical tests. Safety endpoints are occurrence, frequency, and seriousness of Adverse Events (AEs). An independent data safety monitoring board (DSMB) reviewed unblinded data. The trial complies with Good Clinical Practice. Safety endpoints are evaluated descriptively. Comparisons against norm data was done using t-test.

**Results:**

Twenty subjects were randomised, they had very low HRQoL compared to norm data. Mean (SD) PF 31.75 (19.55) (95% Confidence interval; 22.60–40.90) vs 83.5 (23.9) p < 0.001 in Rand-36 PF and mean 0.00 (0.00) in RP. Very low physical performance compared to norm data. 6MWT 442 (180) (95% CI 358–525) vs 662 (18) meters p < 0.001. 31 AEs occurred in 60% of subjects. In 20 AEs, there were at least a possible relationship with the study drug, most commonly cough and chest pain/discomfort.

**Conclusions:**

An (unexpectedly) high frequency of AEs was observed but the DSMB assessed HBO_2_ to have a favourable safety profile. Our data may help other researchers in designing trials.

*Trial Registration*

ClinicalTrials.gov: NCT04842448. Registered 13 April 2021, https://clinicaltrials.gov/ct2/show/NCT04842448. EudraCT: 2021-000764-30. Registered 21 May 2021, https://www.clinicaltrialsregister.eu/ctr-search/trial/2021-000764-30/SE

**Supplementary Information:**

The online version contains supplementary material available at 10.1186/s12879-023-08002-8.

## Background

With more than 500 million confirmed cases of COVID-19 and 10% of infected individuals suffering persistent symptoms, patient-reported low health related quality of life has become a vast problem for individuals, health care systems and society for years to come [1].

Post COVID condition (PCC), also known as Long COVID is commonly defined as having a history of probable or confirmed SARS-CoV-2 infection, and persistent symptoms 3 months from the onset of COVID-19 [2]. Common symptoms are fatigue, shortness of breath and cognitive dysfunction [3]. Mechanisms are still an enigma but suggested mechanisms include auto-immune disease such as dysregulated T-cell activation, chronic oxidative stress, mitochondrial dysfunction, and endothelial dysfunction [4].

No effective evidence-based treatment options for the underlying condition have been widely adopted in clinical practice and many patients seek expensive “remedies” for self-management [5]. Hyperbaric oxygen (HBO_2_) is a possibly effective drug but has not been evaluated for safety and efficacy in compliance with International Conference on Harmonisation of technical requirements for registration of pharmaceuticals for human use-Good Clinical Practice (ICH-GCP) for PCC in clinical trials. It has been suggested to be effective in similar conditions such as Fibromyalgia and Chronic Fatigue syndrome [6, 7]. HBO_2_ has become increasingly popular off-label, a couple of case reports/series are published and RCTs are on the way [8–10]. Since the first submission of this manuscript one RCT including 73 subjects have shown an improvement of neurocognitive function and symptoms in PCC with 40 sessions of HBO_2_ at 2 Bar for 90 min with five-minute air breaks every 20 min [11]. Our hypothesis for use of HBO_2_ in PCC is based on the hyperoxic-hypoxic paradox with activation of Hypoxia Inducible Factor 1 and 2 (HIF-1 and HIF-2) and downstream regulation of hypoxia and inflammatory pathways [12, 13]. The rationale for using fewer and less frequent sessions is based on the above hypotheses, previous clinical experience and from experimental research including own unpublished data. The safety profile of HBO_2_ is well known for accepted indications but has not been described in compliance with ICH-GCP and is not an accepted treatment for patients diagnosed with PCC [14]. The aim of the interim analysis was to evaluate safety of HBO_2_ for our cohort by evaluating reported adverse events (AE) and serious adverse events (SAE) in compliance with ICH-GCP.

## Methods

### Trial design, setting, participants, and interventions

Prospective randomised, parallel arms, placebo-controlled, double blind, clinical trial at Karolinska University Hospital, Sweden.

**Fig. 1:**
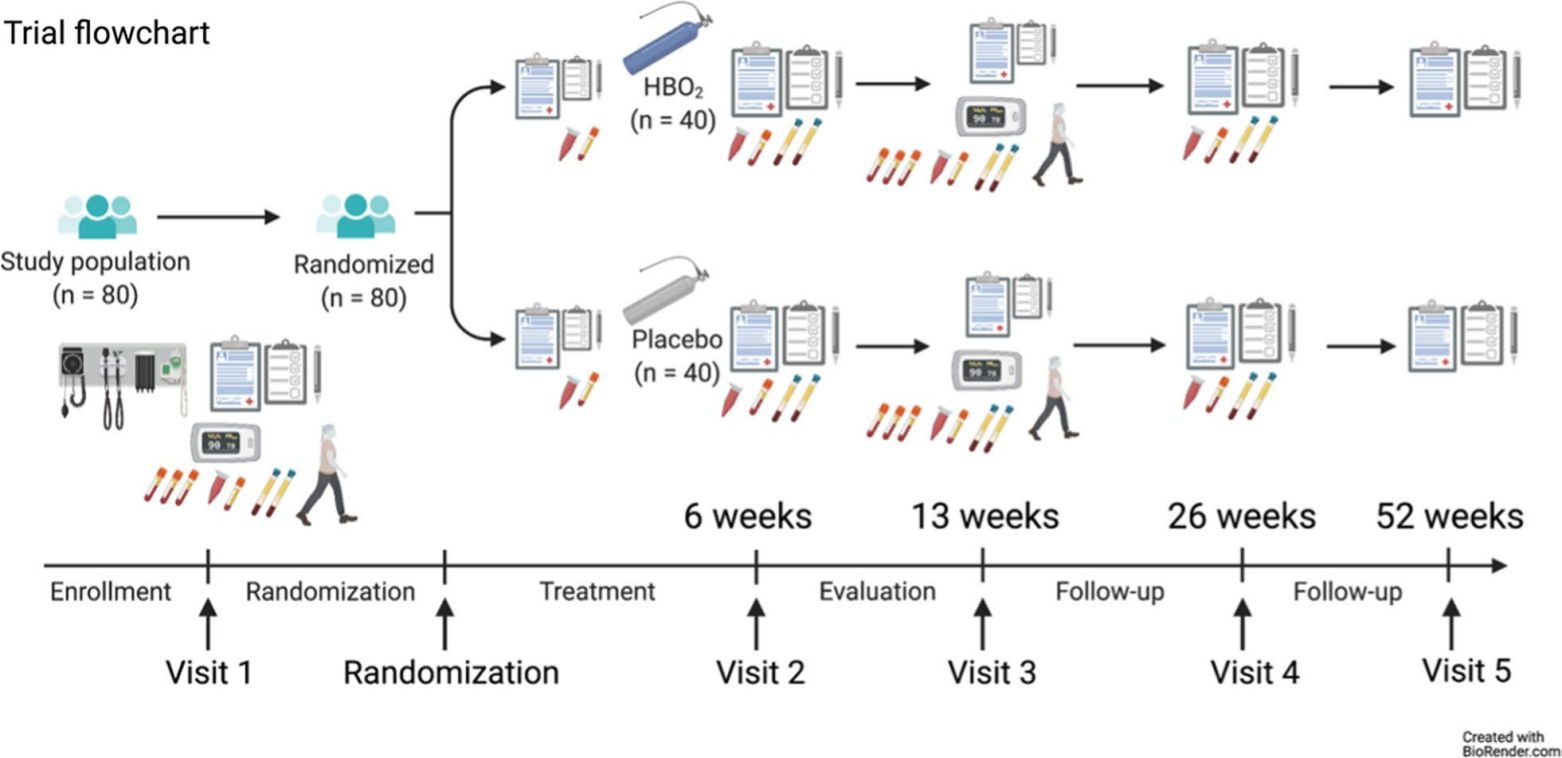
Trial flowchart of the HOT-LoCO trial

**Fig. 2:**
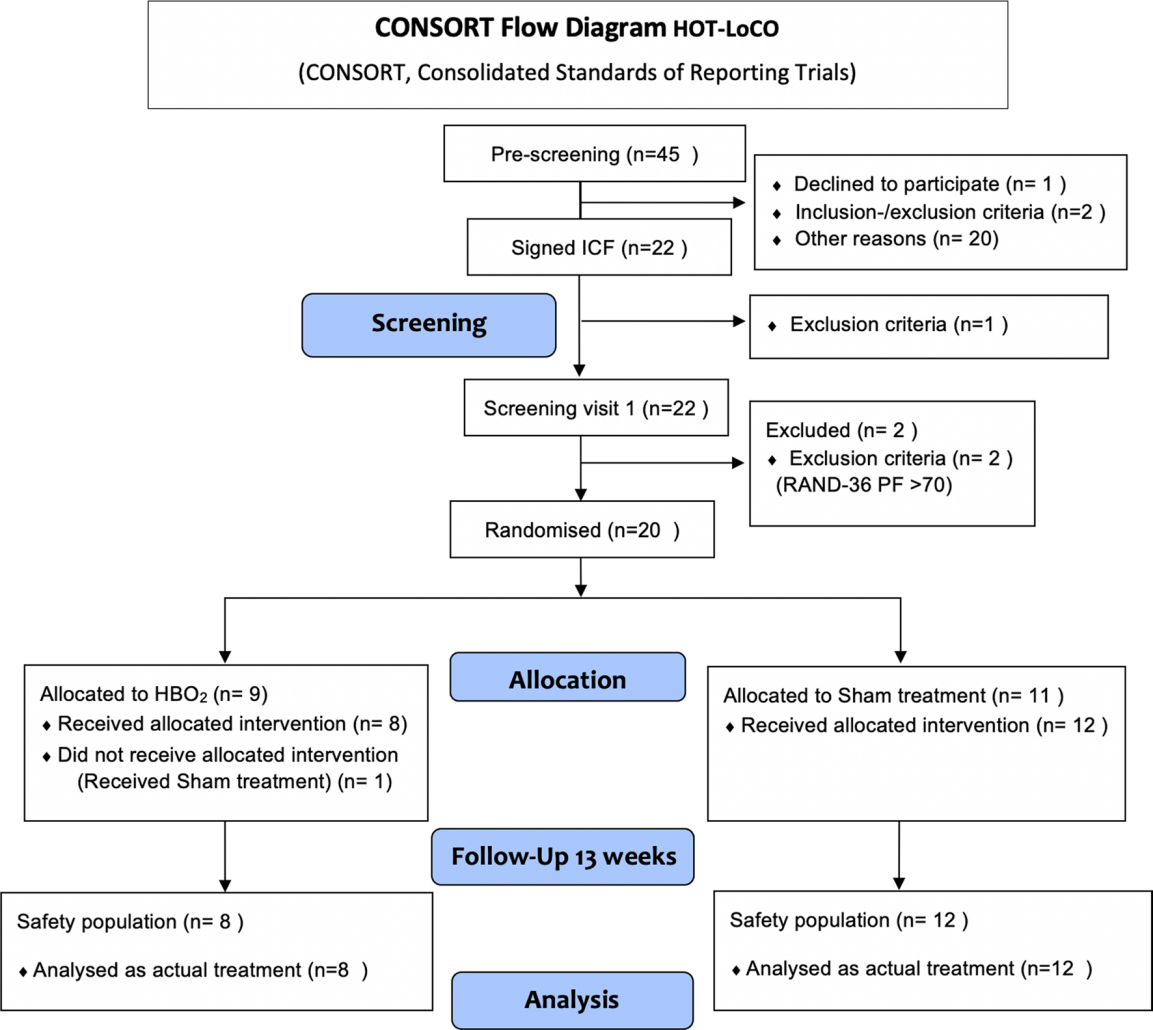
CONSORT Flow diagram of the Safety analysis

We plan to enroll 80 previously healthy subjects diagnosed with PCC (U09.9) randomised (1:1) to HBO_2_ or placebo (sham treatment), maximum ten treatments within 6 weeks from randomisation (Fig. 1). HBO2 was administered at 2.4 Bar for 90 min with two five minutes air breaks. Sham treatment with air was administered by increasing pressure to 1.35 Bar and then reduce to 1.2 Bar. All treatments were given in monoplace chambers (Sechrist, USA). The trial adheres to Consolidated Standards of Reporting Trials (CONSORT) guidelines [15]. The first subject was enrolled September 4 2021 and the interim safety analysis was conducted according to protocol when 20 subjects were followed up 13 weeks, April 28 2022. The CONSORT flow diagram shows the progress of the trial, allocation to treatment according to randomisation. One subject received the wrong allocation and has been removed from the group it guessed, this subject guessed HBO_2_ (Fig. 2). The protocol includes a detailed description and rationale for the primary and main secondary endpoints, including patient reported outcomes (PRO) in line with Standard Protocol Items: Recommendations for Interventional Trials (SPIRIT) SPIRIT-PRO Extension Guidelines [16]. The protocol is available with open access [10].

### Randomisation and blinding

Eligible subjects were randomised in a 1:1 allocation, stratified by disease severity in relation to RAND-36 and gender in blocks (blinded to all study personnel) to either HBO_2_ or Placebo. A computer-based generated randomisation tool (randomizer.at) is used and only delegated staff specifically involved in the treatment and an unblinded monitor have access to the code. The placebo protocol is well established, and even experienced divers cannot differ between “sham treatment” and HBO_2_ [17].

### Endpoints

Primary endpoints are physical domains Physical functioning (PF) and Role Physical (RP) in RAND-36 at 13 weeks. Secondary endpoints are the objective physical tests 6-min walk test (6MWT) and 30 s chair stand (CST), EQ-5D and Reactive hyperemia Index (RHI) at 13 weeks. Safety endpoints are occurrence, frequency, and seriousness of Adverse Events (AE) [10].

### Statistical analysis

Safety endpoints are evaluated descriptively. The number and percentage of patients reporting AEs, and the number of AEs reported are presented. Listings with the events tabulated by system organ class and preferred term are available as supplementary material (Additional file 1).

The number of patients experiencing an AE compared descriptively between groups. All patients with AEs listed individually with subject number in addition to type of event, start and stop time, duration, seriousness, severity, action taken, relationship to trial drug and outcome of AE was presented to the members of the DSMB.

Baseline characteristics of the first 20 subjects are presented as mean ± standard deviation (SD) or number (n) and fraction (%) (Table 1).

**Table 1:** Demographic characteristics for the safety analysis cohort (N = 20)

Female sex	18 (90%)
Body mass index (BMI)	23.3 (7.27)
Ex-smoker	4 (20%)
Never smoker	16 (80%)
Work/study before COVID-19 (%)	95.25 (17.13)
Work/study at baseline (%)	25.0 (36.27)
Education post 2nd, 3 years or more	17 (85%)
Physical activity (min/week)	145.5 (128.2)
Fully vaccinated	13 (65%)
Months since COVID-19 onset	17.15 (1.599)
Positive SARS-CoV-2 antibody	8 (40%)

Baseline data is compared with available norm data from reference populations in Sweden for RAND-36 and EQ-5D and published international reference values for 6MWT and 30 s CST [18]. For RAND-36 the mean of a Swedish reference group with mean age of 57 (20.1) (n = 3422) has been used without adjustment for age and sex [19]. For comparison of EQ-5D the mean of age and sex matched reference values have been used. For RHI no age and sex matched reference data was found and therefore compared with a healthy reference population with mean age 48 [14, 20]. Statistical data analyses and graphs were performed using GraphPad Prism 8.4.3.

All data for efficacy continuous endpoints at baseline are presented using mean, SD, and 95% confidence interval. Comparisons against norm data was done using unpaired t-test.

No adjustment for multiplicity was done since results are descriptive. A p value < 0.05 was considered statistically significant. All reported p values are two-sided. All bar graphs are presented as mean and CI. Significant difference I presented as: *p < 0.05; **p < 0.01; ***p < 0.001.

### Safety and adverse events

Collection of Adverse events (AE) and Serious Adverse Events (SAE) data was started directly after inclusion and recorded until Visit 3. Only SAE was collected outside the treatment period (after Visit 2). Ongoing AE and SAE at the end of Visit 3 will be followed up during long-term follow-up until the subject’s last visit. The definition, handling, follow-up, and reporting of AEs are defined in the original protocol (pp. 34–38). The safety endpoints were evaluated by an independent Data Safety Monitoring Board (DSMB) in the context of the trial design and currently existing information about Long COVID and HBOT. The DSMB is composed of three experts in their respective disciplines of medicine, clinical trial methodology and conduct. The DSMB reviewed the data at the predetermined interim analysis of 20 subjects with safety data available. A charter delineating their guidelines for operating and rules for terminating individual subjects, a portion of or the full trial prematurely was drawn up and agreed upon before the trial started. The members of the DSMB, meeting plan and responsibilities are specified in the original protocol (pp. 6 and 44). The DSMB meeting consists of two parts: An open part where the principal investigator and monitor summarised current status and experience form the trial. During the second, closed part, only the DSMB members discussed safety data (Additional file 2, protocol fr*om DSMB meeting*). The DSMB-members had access to data one week before the meeting and had a dialogue with the senior statistician. All study personnel that participate in the assessment of symptoms and objective findings are blinded to the allocated treatment and have only accessed baseline data and AEs for the whole group.

### Current trial status

The first subject was included in September 2021. 40 subjects have been randomised and 28 have completed 13 weeks follow-up (Visit 3) by December 25, 2022. The second interim analysis will be performed when 40 subjects have completed Visit 3, according to current plan, Q1 2023.

## Results

**Fig. 3:**
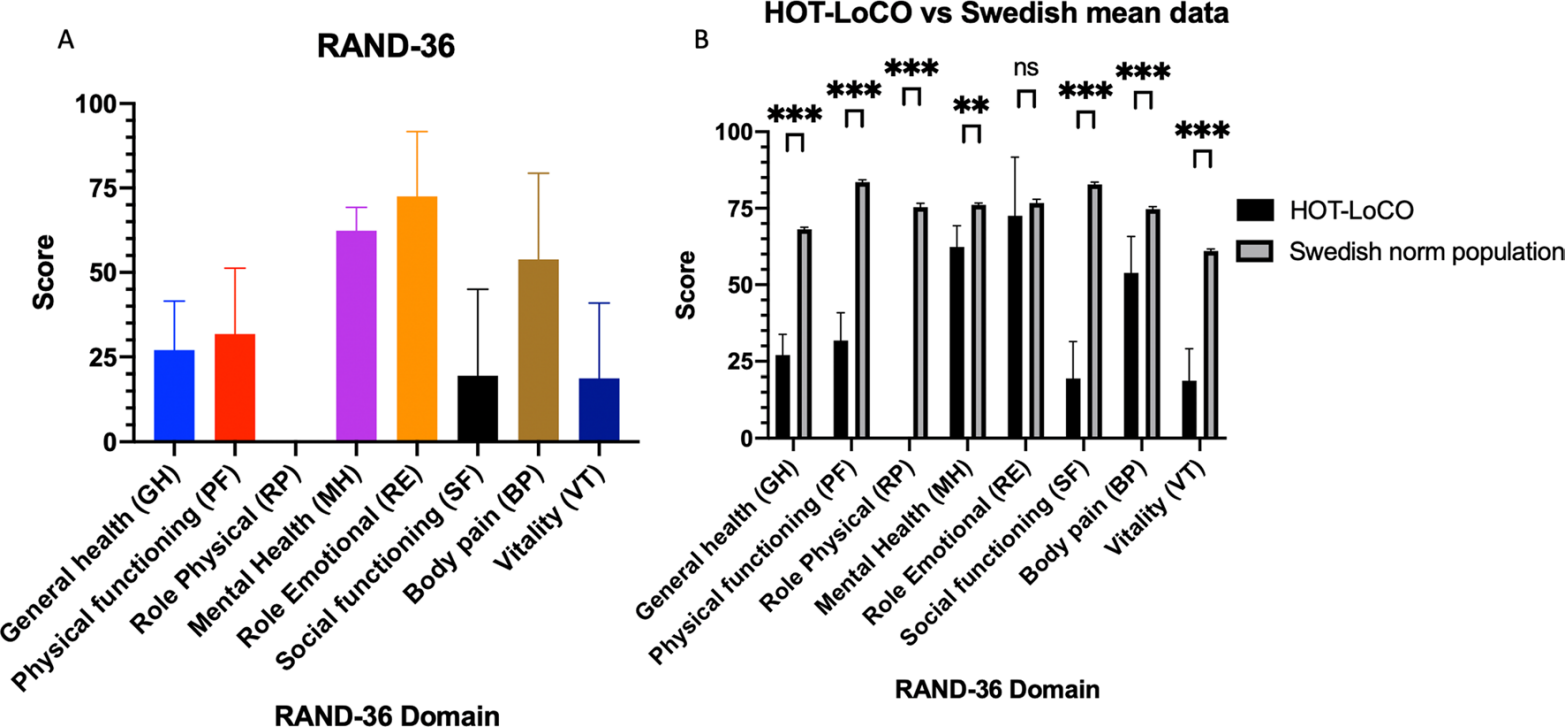
Baseline RAND-36 was very low in Physical domains PF and RP (**A**) and all domains except RE (**B**) compared to a Swedish reference population. Results presented as mean and SD

**Fig. 4:**
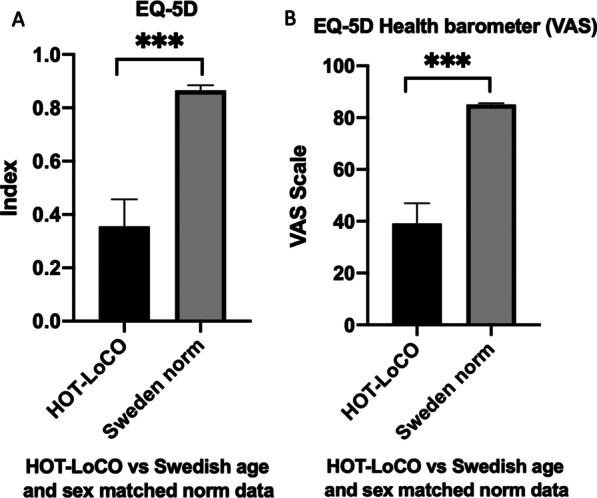
Baseline EQ-5D was very low in index (**A**) and VAS (**B**) compared to Swedish age and sex matched norm data

**Fig. 5:**
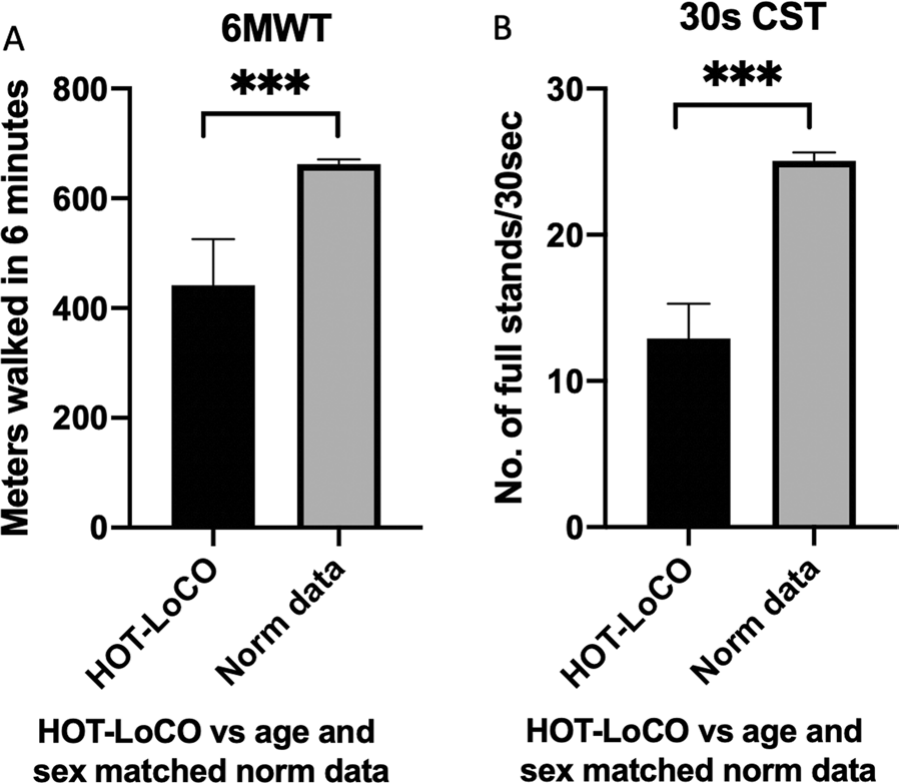
Baseline 6MWT (**A**) and 30 s CST (**B**) was very low compared to international age and sex matched norm data

**Fig. 6:**
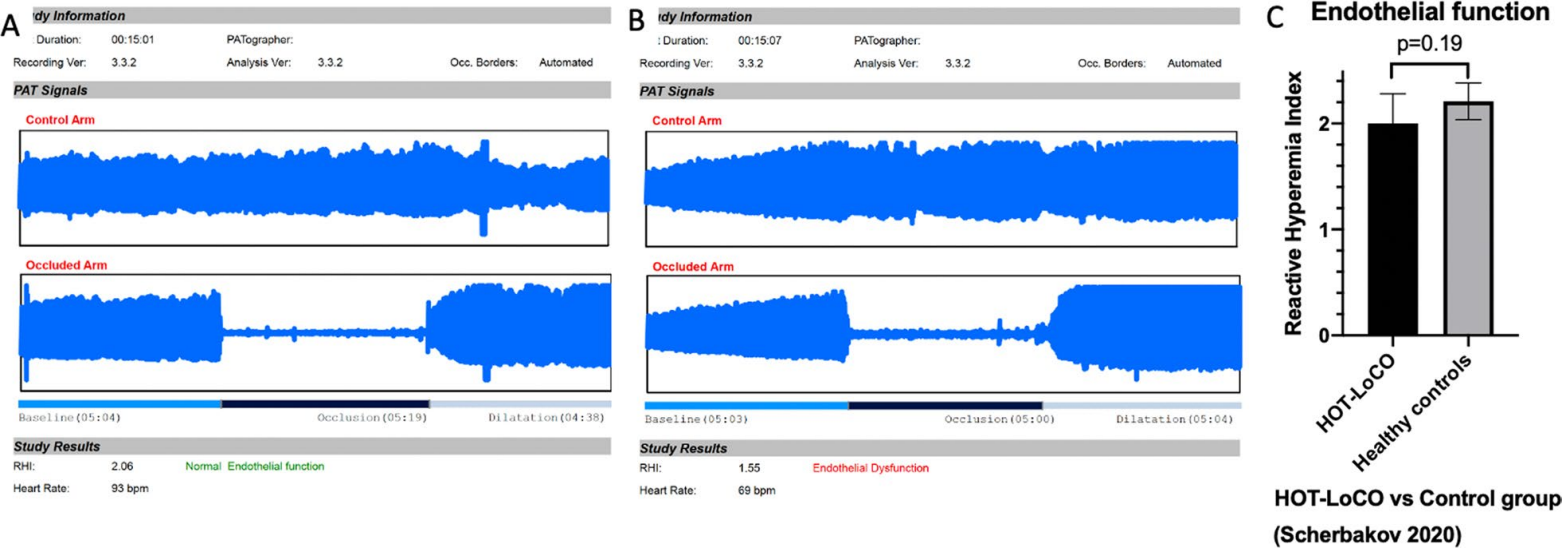
Typical recordings of RHI measurements of ED (**A**) vs normal endothelial function (**B**) and Baseline RHI compared to an older control group (**C**). **A** and **B** shows typical recordings of the EndoPAT 2000 measurement, the “control arm” is measured on the right index finger and “occluded arm” is measured on the left index finger. **C** Shows mean and SD of RHI in our cohort compared to a previously published control group (n = 20) with mean age 48 ± 14 (Scherbakov 2020)

Twenty subjects had safety data available at 13 weeks. Demographic characteristics are presented in Table 1. Self-reported HRQoL in RAND-36 was very low in physical domains at baseline compared to Swedish norm data; PF 31.75(19.55) vs 83.5(23.9) (95% Confidence interval 22.60–40.90) p < 0.001, RP 0(0) vs 75.4(37.6) p < 0.001 and statistically significantly lower in all domains except Role emotional (RE) (Fig. 3). Self-reported HRQoL in EQ-5D was very low; index 0.36 (0.22) (95% CI 0.25–0.46) vs 0.87(95% CI 0.82–0.92) p < 0.001 and visual analogue scale (VAS) 39.1 (16.75)) (95% CI 31.26–46.94) vs 85.1 (1.05)) (95% CI 81–89) p < 0.001 compared to age and sex matched norm data (Fig. 4). Performance in physical tests were very low at baseline compared to international norm data; 6MWT 442 (180) (95% CI 357.7–525.8) vs 662 (18) meters and CST 13(5.1) (95% CI 10.51–15.29) vs 25 (1.23) (95% CI 22.95–27.60) stands in 30 s (Fig. 5). Baseline data of RHI in the 20 subjects in the interim analysis; 35% of the subjects have RHI < 1.67 i.e., endothelial dysfunction and 30% RHI 1.67–2.10 i.e., borderline ED at baseline. While numerically lower, this did not reach statistical significance compared to a ten-year older control group (Fig. 6).

Thirty-one AEs were recorded, at least one in 60% of subjects. No SAE was reported. Most AE were grade 1, 6 were grade 2. In 20 AEs, there was at least a possible relationship with the study drug. The most common AE was cough and chest pain/discomfort. All AE were transient. (Additional file 1, AE listings).

## Discussion

Our results show that the self-reported HRQoL is extremely low in our cohort compared to previously published data on Long COVID [21]. The reference data for RAND-36 and EQ-5D are based on Swedish populations matched for age and sex but not adjusted for level of education. Our cohort consists of highly educated subjects, therefore HRQoL is expected to be even higher but may also explain the very low result of Role Physical (expectations). Most of our subjects were infected during the first wave 2020 and were unvaccinated at the time. There may be a selection bias depending on this fact and there has also been a selection of patients referred to our PCC clinic; only the most serious cases were accepted. According to our protocol with “sham treatment” we are not able to adjust the dose with pressure, only time. For some subjects the time was reduced due to cough or chest discomfort during treatment and some subjects was not able to complete all ten treatments. A protocol that allows a lower pressure or individually adjusted may be beneficial for compliance. The previously published RCT reported no significant difference in side effects between the groups (35.1% and 38.9%, p = 0.739 in the HBO_2_ and control groups respectively) and no discontinuation of the treatment due to side effects [11]. Given the frailty of this group it’s possible that AEs occurred in the placebo treatment group due to the effort of participation or by breathing non humidified air. Alternative explanations for the higher rate of adverse events are difference in disease severity or difference in treatment protocols. Very few in our cohort would be able to accept treatments on two consecutive days, due to severe fatigue.

## Conclusions

HBO_2_ appears to have a favorable safety profile for PCC considering the absence of SAE but an unexpected high frequency of AE was observed. Most of them were mild and all of them were transient. We speculate that frequency of AE could be reduced by individual dosing. This safety analysis enables further investigation of the efficacy of HBO_2_ within the HOT-LoCO trial and may help other researchers in designing trials.

## Supplementary Information

**Additional file 1.** AE listing, AE Interim 1 Safety report.pdf.

**Additional file 2.** Protocol from DSMB meeting, DSMB protocol 2022-05-09.pdf.

## Data Availability

An anonymised list of adverse events and the DSMB protocol is available as additional files. The full protocol is available with open access [10]. Anonymised lists of baseline data (blinded to intervention) on subject level will be available upon reasonable request. A full description of the intended use of the data must be sent to the corresponding author for review and approval. Participant consent for data sharing is conditioned and new ethics approval may be required.

## Abbreviations

PCC: Post COVID condition
HRQoL: Health related quality of life
HBO2: Hyperbaric oxygen
RAND-36: RAND-36 Questions Questionnaire for HRQoL
PF: Physical functioning domain of RAND-36
RP: Role physical domain of RAND-36
AE: Adverse event
SAE: Serious adverse event
6MWT: Six Minute Walk Test
30 s CST: 30 Seconds Chair Stand Test
EQ-5D: EuroQol Group 5 Questions Questionnaire for HRQoL
RHI: Reactive Hyperemia Index (measurement of endothelial function)
ICH-GCP: International Council for Harmonisation-Good Clinical Practice
DSMB: Data Safety Monitoring Board
VAS: Visual Analogue Scale
