# Supplementary material for: Hyperbaric oxygen therapy for long COVID (HOT-LoCO), an interim safety report from a randomised controlled trial
Source: BMC Infect Dis. 2023 Jan 20;23:33. doi: 10.1186/s12879-023-08002-8 (PMC9854077; doi:10.1186/s12879-023-08002-8)
Supplement: Supplementary file 1 — Additional file 1. AE listing, AE Interim 1 Safety report.pdf. [file 12879_2023_8002_MOESM1_ESM.pdf]

Additional file 1
AE listing

MedDRA code	MedDRA PT	MedDRA SOC	Severity (CTCAE grade)	Relationship to IMP
10014020	Ear pain	Ear and labyrinth disorders	Grade 1 - Mild	Unlikely
10062352	Respiratory tract infection	Respiratory, thoracic and mediastinal disorders	Grade 1 - Mild	Unlikely
10003658	Atrial fibirillation	Cardiac disorders	Grade 1 - Mild	Unlikely
10003988	Back pain	Musculoskeletal and connective tissue disorders	Grade 2 - Moderate	Unlikely
10008479	Chest pain	General disorders and administration site conditions	Grade 2 - Moderate	Possible
10052137	Ear discomfort	Ear and labyrinth disorders	Grade 1 - Mild	Unlikely
10013968	Dyspnoea	Respiratory, thoracic and mediastinal disorders	Grade 2 - Moderate	Unlikely
10062352	Respiratory tract infection	Respiratory, thoracic and mediastinal disorders	Grade 1 - Mild	Unlikely
10040753	Sinusitis	Infections and infestations	Grade 1 - Mild	Unlikely
10028411	Myalgia	Musculoskeletal and connective tissue disorders	Grade 1 - Mild	Possible
10011224	Cough	Respiratory, thoracic and mediastinal disorders	Grade 1 - Mild	Possible
10011224	Cough	Respiratory, thoracic and mediastinal disorders	Grade 2 - Moderate	Probable
10011224	Cough	Respiratory, thoracic and mediastinal disorders	Grade 1 - Mild	Probable
10011224	Cough	Respiratory, thoracic and mediastinal disorders	Grade 1 - Mild	Possible
10011224	Cough	Respiratory, thoracic and mediastinal disorders	Grade 1 - Mild	Probable
10011224	Cough	Respiratory, thoracic and mediastinal disorders	Grade 1 - Mild	Probable
10011224	Cough	Respiratory, thoracic and mediastinal disorders	Grade 1 - Mild	Probable
10011224	Cough	Respiratory, thoracic and mediastinal disorders	Grade 1 - Mild	Probable
10011224	Cough	Respiratory, thoracic and mediastinal disorders	Grade 1 - Mild	Probable
10011224	Cough	Respiratory, thoracic and mediastinal disorders	Grade 1 - Mild	Probable
10047571	Visual impairment	Eye disorders	Grade 2 - Moderate	Probable
10084268	COVID-19	Infections and infestations	Grade 2 - Moderate	Unlikely
10011224	Cough	Respiratory, thoracic and mediastinal disorders	Grade 1 - Mild	Possible
10011224	Cough	Respiratory, thoracic and mediastinal disorders	Grade 1 - Mild	Probable
10011224	Cough	Respiratory, thoracic and mediastinal disorders	Grade 1 - Mild	Probable

10011224	Cough	Respiratory, thoracic and mediastinal disorders	Grade 1 - Mild	Probable
10011224	Cough	Respiratory, thoracic and mediastinal disorders	Grade 1 - Mild	Probable
10062352	Respiratory tract infection	Repiratory, thoracic, and mediastinal disorders	Grade 1 - Mild	Unlikely
10011224	Cough	Respiratory, thoracic and mediastinal disorders	Grade 1 - Mild	Probable
10008469	Chest discomfort	General disorders and administration site conditions	Grade 1 - Mild	Probable
10062352	Respiratory tract infection	Respiratory, thoracic and mediastinal disorders	Grade 1 - Mild	Unlikely
