# Supplementary material for: Hyperbaric oxygen therapy for long COVID (HOT-LoCO), an interim safety report from a randomised controlled trial
Source: BMC Infect Dis. 2023 Jan 20;23:33. doi: 10.1186/s12879-023-08002-8 (PMC9854077; doi:10.1186/s12879-023-08002-8)
Supplement: Supplementary file 2 — Additional file 2. Protocol from DSMB meeting, DSMB protocol 2022-05-09.pdf. [file 12879_2023_8002_MOESM2_ESM.pdf]

APPENDIX I – DSMB EVALUATION REPORT TEMPLATE

DSMB EVALUATION REPORT

Sponsor:	Type of Meeting:	Meeting Date:
Karolinska University Hospital	☑ VL ☐ Face to face meeting	2022-05-09
**Protocol #:**	**Evaluation Report No.:**	
2021-000764-30	☑ 1 ☐ 2 ☐ 3 ☐ 4 ☐ 5 ☐ 6	

DSMB Attendees, closed part of meeting:			
<DSMB Chairperson>	☑ Yes<br>☐ No	External expert	☐ Yes<br>☑ No
<DSMB Member 2>	☑ Yes<br>☐ No	External expert	☐ Yes<br>☑ No
<DSMB Member 3>	☑ Yes<br>☐ No	Statistician	☐ Yes<br>☑ No
No conflict of interest statement identified by the Chairperson:			☑ Yes<br>☐ No
DSMB Quorum fulfilled (all 3 DSMB permanent members present):			☑ Yes<br>☐ No

The DSMB for the clinical trial HOT-LOCO 2021-000764-30 reviewed the Data Report No.			
☑ 1 -	☐ 4 -		
☐ 2 -	☐ 5 -		
☐ 3 -	☐ 6 -		
**Summary of discussion of the meeting:**	All DSMB-members have thoroughly scrutinized the provided and appropriate listings and tables (see below) covering the, so far, included subjects.<br><br><u>Conclusion:</u> The unblinded data provided to the DSMB-members raise no safety concerns. We do not envisage any reason to discontinue the study. The study can be continued as planned based on safety issues.		

Outcome of DSMB Meeting:		
As a result, the DSMB recommends proceeding with the next planned trial part:		☑ Yes ☐ No
**Reason:**	No safety concerns raised	
**Additional Comments:**	The study appears to be conducted in accordance with GCP statutes. Questions to the statistician and the study team have been addressed. They are all clarified without any hesitation or delay.	

DSMB Chairperson:		
Printed name: Kjell Ahlén	Signature: [signature]	Date: 13 May 2022

Listings and tables that were provided for review:
Listings 9.2.4.1. Adverse Events, Interim 1 unblinded (SAF)
Listing 9.2.1.2 Inclusion and Exclusion Criteria
Listing 9.2.1.6.1 Demographics
Listing 9.2.1.6.2 Demographics - Living Conditions.
Listing 9.2.1.7.1 Baseline Characteristics (SAF).
Listing 9.2.1.7.2 Baseline Characteristics RAND-36 (SAF).
Listing 9.2.1.7.3 Baseline Characteristics EQ-5D (SAF).
Listing 9.2.1.7.4 Baseline Characteristics Other Endpoints (SAF).
Listing 9.2.1.7.5 Baseline Characteristics Nexfin (SAF).

Table 1. AE Overview
Table 8.1.2 Summary Statistics: Violation of Inclusion and Exclusion Criteria (SAF).
Table 8.1.4.1 Summary Statistics: Demography (SAF).
Table 8.1.4.2 Summary Statistics: Demographic Characteristics, Continuous Variables (SAF).
Table 8.1.5.1 Summary Statistics: Baseline Characteristics, Covid Symptoms (SAF).
Table 8.1.5.2 Summary Statistics: Baseline Characteristics, Living Conditions (SAF).
Table 8.1.5.3 Summary Statistics: Baseline Characteristics, Covid-19 tests (SAF).
Table 8.1.5.4 Summary Statistics: Baseline Characteristics, Primary Endpoints (SAF).
Table 8.1.5.5 Summary Statistics: Baseline Characteristics, Main Secondary Endpoints (SAF).
Table 8.1.5.6 Summary Statistics: Baseline Characteristics, Endothelial Function (SAF).
Table 8.2.2.1 Summary of Adverse Events, (SAF).
Table 8.2.2.3 Summary Adverse Events by SOC, PT and Treatment Group, (SAF).
